# The Association of Body Fat Percentage With Hypertension in a Chinese Rural Population: The Henan Rural Cohort Study

**DOI:** 10.3389/fpubh.2020.00070

**Published:** 2020-03-20

**Authors:** Ruiying Li, Zhongyan Tian, Yanhua Wang, Xiaotian Liu, Runqi Tu, Yan Wang, Xiaokang Dong, Yikang Wang, Dandan Wei, Huiling Tian, Zhenxing Mao, Linlin Li, Wenqian Huo, Chongjian Wang, Ronghai Bie

**Affiliations:** ^1^Department of Epidemiology and Biostatistics, College of Public Health, Zhengzhou University, Zhengzhou, China; ^2^Department of Prevention and Health Care, Military Hospital of Henan Province, Zhengzhou, China

**Keywords:** hypertension, body fat percentage, dose–response relationship, prevalence, Chinese rural population

## Abstract

**Background:** Obesity is an important risk factor for hypertension. Previous studies have explored the association between body fat percentage (BFP) and hypertension, but evidence on the consistency of the association remains uncertain and limited. The aim of this study was to explore the relationship between BFP and hypertension in a Chinese rural population.

**Methods:** The present cross-sectional study including 38,913 eligible individuals was conducted in rural areas of Henan province. BFP was measured by bioelectrical impedance methods using Omron body fat and weight measurement device. Logistic regression models and restricted cubic spline regression models were performed to investigate the relationship between BFP and hypertension. Receiver operating characteristic (ROC) analyses were used to compare the discriminating power of adiposity indices.

**Results:** The age-standard prevalence of hypertension was 23.74 and 17.87% in males and females, respectively. Compared with the first quartile of BFP, the adjusted odds ratios (ORs) and 95% confidence intervals (CIs) for hypertension in the highest BFP quartile were 3.30 (95% CI: 2.85, 3.83) in males and 2.66 (95% CI: 2.36, 2.99) in females, and the adjusted ORs increased along with increasing BFP levels. The areas under ROC and 95% CIs of BFP were 0.673 (0.665, 0.682) in males and 0.696 (0.689, 0.703) in females, respectively.

**Conclusions:** BFP was significantly positively associated with the prevalence of hypertension in both males and females in the Chinese rural population. Controlling of body fat should be emphasized in rural areas of China.

**Clinical Trial Registration:** Registration number: ChiCTR-OOC-15006699. http://www.chictr.org.cn/showproj.aspx?proj=11375

## Introduction

Hypertension has become a significant health problem due to its rising prevalence and association with other diseases (1, 2). Studies indicated that the percentage of Chinese adults with hypertension increased from 20 to 31.2%in men and from 17 to 28.0% in women between 2002 and 2012 (3, 4). The prevalence of obesity, an important risk factor for chronic diseases, has also significantly increased over the last few decades (5, 6). For instance, a prior study including nearly 50,000 Chinese adults revealed that between 1993 and 2009, the prevalence of general obesity approximately tripled in males and doubled in females, that is, from 2.9 to 11.4% and from 5.0 to 10.1%, respectively (5).

Numerous studies have confirmed that obesity could increase the risk of hypertension (7, 8). For instance, a recent study published in 2019 reported that obesity was highly associated with hypertension (9) and was responsible for more than half of essential hypertension events (10). Early identification and control of individuals suffered from hypertension were, therefore, critical for improving the quality of life and reducing mortality in a high-risk population. Body fat percentage (BFP), as a more accurate and reliable indicator for evaluating general obesity compared to BMI, cannot be neglected (11, 12). Briefly, a previous study performed in southern Brazil showed that compared to individuals with lower BFP, individuals who were with higher BFP were prone to have a greater risk of hypertension (13). Moreover, Jiang et al. also reported that BFP was a better indicator for discriminating hypertension, regardless of gender (14). Recently, research conducted in South Korea indicated that increased BFP was associated with the high risk of hypertension even in non-obese subjects (12). However, Alvin Chandra et al. found that neither total nor subcutaneous adiposity was related to the onset of hypertension (15).

In summary, although some studies have explored the relationship between BFP and prevalent hypertension, evidence related to the actual association remains inconsistent and even controversial. Moreover, to date, information about this relationship in rural areas of China is also yet unclear. Hence, the aim of this study was to explore the relationship between BFP and risk of hypertension in the Chinese rural population.

## Methods

### Study Design

The Henan Rural Cohort Study (Registration number: ChiCTR-OOC-15006699), which was established in five rural areas of Henan province since July 2015, aimed to evaluate the prevalence as well as the risk factors of cardiometabolic disease in the Chinese rural population, as described previously (16, 17). Briefly, the study samples were selected as follows. First, via simple cluster sampling, we selected five rural counties, that is, Tongxu, Yima, Suiping, Xinxiang, and Yuzhou, respectively. Second, one to three townships were further selected based on some factors such as the compliance of the residents and medical conditions. After that, except for permanent residents with a severe physical or mental disease, those aged 18–79 years in the selected townships were enrolled into the large-scale study. A total of 39,259 people were included and signed the written informed consent. In the present study, individuals were excluded if they were missing information regarding BFP (*n* = 323) or diagnosis of hypertension (*n* = 23). As a result, 38,913 eligible individuals were retained for the present analysis. For the current study, approval of Zhengzhou University Life Science Ethics Committee was required, and written informed consent was obtained from all study subjects. In addition, this survey was also carried out in compliance with the guidelines of the Helsinki Declaration.

### Data Collection and Anthropometric Measurements

Demographic characteristics, lifestyle behaviors, as well as the personal and family history of diseases were collected for each individual by well-trained investigators via face-to-face interviews. On the same day, anthropometric measurements such as blood pressure, body height, weight, and BFP were also collected by trained interviewers using calibrated equipment and according to standard operating procedure.

With a prohibition of taking exercise, smoking, as well as drinking caffeine for above half an hour, the blood pressure of all participants was determined using an electronic sphygmomanometer (HEM-770AFuzzy; Omron, Japan) and measured in three consecutive measurements together with a 30-s interval after above 5 min of rest; the average of three readings, after that, was calculated for analyses (8, 18). Body height, waist circumference (WC), hip circumference (HC), and weight were measured twice to the nearest 0.1, 0.1, 0.1 cm, and 0.1 kg, respectively, with the staff wearing light clothing and barefoot. Likewise, the average readings were used for analyses. In addition, Omron body fat and weight measurement device (V-body HBF-371; Omron, Kyoto, Japan), which relied on bioelectrical impedance analysis method, was determined to measure BFP and visceral fat index (VFI) in this study (19). Calculation of other adiposity measures was based on the following formulas: body mass index (BMI) = weight (kg)/[height (m)]^2^; waist-to-hip ratio (WHR) = WC (cm)/HC (cm); waist-to-height ratio (WHtR) = WC (cm)/height (cm).

### Assessment of Potential Covariates

Education level was classified into three groups, that is, elementary school or below, junior high school, and high school or above. Marital status was grouped into unmarried/divorced/widowed as well as married/cohabitating. Socioeconomic status was evaluated based on the average monthly individual income. Current smoking was defined as smoking one or more cigarette per day for sequential or cumulative over a 6-month period (20). Current drinking was defined as consuming alcohol at least 12 times per year (8). Physical activity, as defined by the international physical activity questionnaire (IPAQ 2001), was classified into low, moderate, and high (21). Individuals whose parents or siblings had a history of hypertensive disorder were recorded as having a family history of hypertension.

### Definition of Hypertension

Individuals with hypertension were defined as those who had a measured average systolic blood pressure (SBP) ≥ 140 mmHg, and/or diastolic blood pressure (DBP) ≥ 90 mmHg, or who already diagnosed with hypertension and currently using antihypertensive drugs (22).

### Statistical Analysis

Continuous variables and categorical variables were presented as means ± standard deviations (SDs) and numbers (percentages), with a corresponding analysis using one-way analysis of variance as well as chi-square test, respectively. The age-standard prevalence of hypertension of this study was assessed according to the 2010 census data of the Chinese adult population. With the aid of the restricted cubic spline regression, which was along with three knots placed at the 25 (reference), 50, and 75th percentiles of BFP levels, respectively, the dose–response relationship of continuous BFP levels with the risk for hypertension could be examined. Binary logistic regression models were used to yield odds ratios (ORs) and 95% confidence intervals (CIs) for hypertension based on BFP quartiles and per 1-SD increment. Model 1 was unadjusted. Model 2 was only adjusted for age. Model 3 additionally adjusted for education level, average monthly individual income, marital status, smoking, alcohol consumption, physical activity, high-fat diet, more vegetable and fruit intake, type 2 diabetes (T2DM), family history of hypertension, triglyceride (TG), cholesterol (TC), fasting blood glucose (FBG), hypersensitive c-reactive protein (Hs-CRP), high-density lipoprotein cholesterol (HDL-C), and low-density lipoprotein cholesterol (LDL-C). Analyses of receiver operating characteristic (ROC) as well as the areas under the curves (AUCs) were conducted to compare the discriminating power for the risk of hypertension in terms of six adiposity indices and the DeLong et al. method (23) was used to test the differences in AUCs.

Statistical analyses of data were performed by SPSS 21.0 and MedCalc software, and figures of restricted cubic spline were produced using SAS 9.1 software package. Two-sided *P* < 0.05 were considered statistically significant.

## Results

### Characteristics of Study Participants

**Figure 1:**
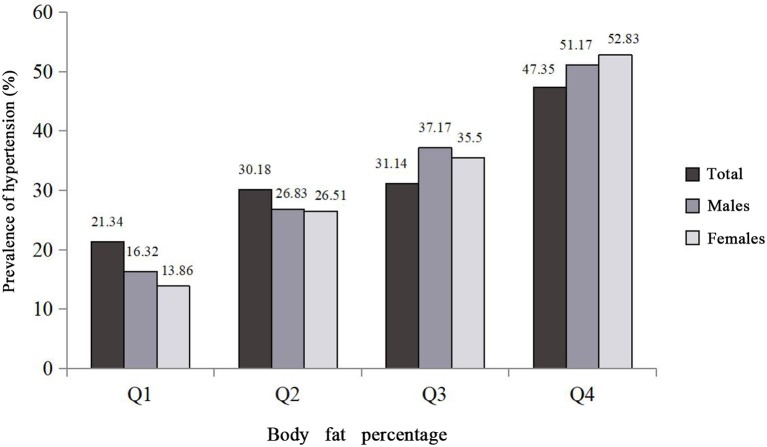
Prevalence of hypertension distributions among the study population and the quartiles of body fat percentage. Body fat percentage was divided into sex-specific quartiles (for total population: Q1: <25.5, Q2: 25.5–30.7, Q3: 30.7–35.1, Q4: ≥35.1; for males: Q1: <21.2, Q2: 21.2–24.6, Q3: 24.7–27.8, Q4: ≥27.9; for females: Q1: <31.0, Q2: 31.0–34.0, Q3: 34.1–36.8, Q4: ≥36.9).

The final pooled study comprised 38,913 individuals, 60.6% of whom were females. The mean age of the total population, males, and females was 55.57 ± 12.18 years, 56.6 ± 12.30 years, and 54.9 ± 12.1 years, respectively. A total of 12,678 cases of hypertension (5,048 males and 7,630 females) were ascertained in the present study. Age-standard prevalence of hypertension for males and females was 23.74 and 17.87%, respectively. Compared to subjects without hypertension, subjects with hypertension had higher age, family history of hypertension, FBG, T2DM, Hs-CRP, TG, TC, LDL-C, BFP, VFI, BMI, WC, WHR, and WHtR, and had lower education level and average monthly individual income and physical activity in the total population, males and females (*P* < 0.001), while marital status in males (*P* = 0.051) and smoking status in females (*P* = 0.850) were not found to be related to hypertension (Table 1). In addition, the prevalence of hypertension gradually increased with the increase of BFP quartiles. In Figure 1, those who were in the highest BFP quartile also had highest hypertension prevalence (47.35% in the total population, 51.17% in males, and 52.83% in females).

**Table 1:** Baseline characteristics of the study participants according to hypertension status.

	**Total (*****n*** **=** **38,913)**			**Males (*****n*** **=** **15,325)**			**Females (*****n*** **=** **23,588)**		
	**Non-hypertension**	**Hypertension**	***P***	**Non-hypertension**	**Hypertension**	***P***	**Non-hypertension**	**Hypertension**	***P***
Age (years), mean ± SD	53.25 ± 12.43	60.36 ± 10.09	<0.001	55.08 ± 12.61	59.65 ± 11.03	<0.001	52.07 ± 12.16	60.83 ± 9.39	<0.001
Education level, *n* (%)			<0.001			<0.001			<0.001
Elementary school or below	10,648 (40.59)	6,747 (53.22)		3,344 (32.54)	1,818 (36.01)		7,304 (45.77)	4,929 (64.60)	
Junior high school	11,154 (42.52)	4,367 (34.45)		4,848 (47.17)	2,243 (44.43)		6,306 (39.52)	2,124 (27.84)	
High school or above	4,433 (16.90)	1,564 (12.34)		2,085 (20.29)	987 (19.55)		2,348 (14.71)	577 (7.56)	
Average monthly individual income, *n* (%)			<0.001			<0.001			<0.001
<500 RMB	8,854 (33.75)	4,993 (39.38)		3,600 (35.03)	1,914 (37.92)		5,254 (32.92)	3,079 (40.35)	
500–1,000 RMB	8,620 (32.86)	4,181 (32.98)		3,257 (31.69)	1,621 (32.11)		5,363 (33.61)	2,560 (33.55)	
≥1,000 RMB	8,761 (33.39)	3,504 (27.64)		3,420 (33.28)	1,513 (29.97)		5,341 (33.47)	1,991 (26.09)	
Marital status, *n* (%)			<0.001			=0.051			<0.001
Married/cohabitation	23,904 (91.11)	11,056 (87.21)		9289 (90.39)	4512 (89.38)		14,615 (91.58)	6544 (85.77)	
Unmarried/divorced/widowed	2,331 (8.89)	1,622 (12.79)		988 (9.61)	536 (10.62)		1,343 (8.42)	1,086 (14.23)	
Smoking			<0.001			<0.001			=0.850
Never smokers, *n* (%)	19,038 (72.57)	9,303 (73.38)		3,139 (30.54)	1,701 (33.70)		15,899 (99.63)	7,602 (99.63)	
Ex-smokers, *n* (%)	1,833 (6.99)	1,316 (10.38)		1,817 (17.68)	1,310 (25.95)		16 (0.10)	6 (0.08)	
Current smokers, *n* (%)	5,364 (20.45)	2,059 (16.24)		5,321 (51.78)	2,037 (40.35)		43 (0.27)	22 (0.29)	
Alcohol consumption			<0.001			<0.001			<0.001
Never drinkers, *n* (%)	20,380 (77.68)	9,699 (76.50)		4,951 (48.18)	2,216 (43.90)		15,429 (96.69)	7,483 (98.07)	
Ex-drinkers, *n* (%)	1,101 (4.20)	700 (5.52)		1,056 (10.28)	681 (13.49)		45 (0.28)	19 (0.25)	
Current drinkers, *n* (%)	4,754 (18.12)	2,279 (17.98)		4,270 (41.55)	2,151 (42.61)		484 (3.03)	128 (1.68)	
Physical activity, *n* (%)			<0.001			<0.001			<0.001
Low	7,776 (29.64)	4,721 (37.24)		3,318 (32.29)	2,102 (41.64)		4,458 (27.94)	2,619 (34.33)	
Moderate	10,368 (39.52)	4,363 (34.41)		2,973 (28.93)	1,301 (25.77)		7,395 (46.34)	3,062 (40.13)	
High	8,091 (30.84)	3,594 (28.35)		3,986 (38.79)	1,645 (32.59)		4,105 (25.72)	1,949 (25.54)	
Family history of hypertension, *n* (%)			<0.001			<0.001			<0.001
No	22,200 (84.62)	9,191 (72.50)		8,935 (86.94)	3,696 (73.22)		13,265 (83.12)	5,495 (72.02)	
Yes	4,035 (15.38)	3,487 (27.50)		1,342 (13.06)	1,352 (26.78)		2,693 (16.88)	2,135 (27.98)	
T2DM, *n* (%)			<0.001			<0.001			<0.001
No	24,405 (93.16)	10,789 (85.26)		9,524 (92.84)	4,387 (87.08)		14,881 (93.37)	6,402 (84.06)	
Yes	1,791 (6.84)	1,865 (14.74)		735 (7.16)	651 (12.92)		1,056 (6.63)	1,214 (15.94)	
FBG (mmol/L), mean ± SD	5.39 ± 1.39	5.85 ± 1.66	<0.001	5.41 ± 1.43	5.78 ± 1.61	<0.001	5.38 ± 1.37	5.89 ± 1.70	<0.001
Hs-CRP, mean ± SD	1.40 ± 2.05	1.53 ± 2.09	<0.001	1.49 ± 2.23	1.50 ± 2.16	=0.811	1.34 ± 1.92	1.54 ± 2.05	<0.001
TG (mmol/L), mean ± SD	1.58 ± 1.05	1.88 ± 1.24	<0.001	1.59 ± 1.10	1.81 ± 1.26	<0.001	1.57 ± 1.03	1.92 ± 1.22	<0.001
TC (mmol/L), mean ± SD	4.66 ± 0.94	4.98 ± 1.05	<0.001	4.56 ± 0.91	4.82 ± 1.03	<0.001	4.72 ± 0.95	5.09 ± 1.04	<0.001
HDL-C (mmol/L), mean ± SD	1.34 ± 0.33	1.29 ± 0.33	<0.001	1.28 ± 0.32	1.23 ± 0.33	<0.001	1.39 ± 0.33	1.33 ± 0.33	<0.001
LDL-C (mmol/L), mean ± SD	2.80 ± 0.79	3.01 ± 0.87	<0.001	2.78 ± 0.78	2.91 ± 0.85	<0.001	2.82 ± 0.80	3.07 ± 0.87	<0.001
BFP, mean ± SD	29.09 ± 6.64	32.06 ± 6.33	<0.001	23.34 ± 5.09	26.35 ± 4.63	<0.001	32.79 ± 4.55	35.84 ± 4.06	<0.001
VFI, mean ± SD	8.55 ± 4.19	11.31 ± 4.81	<0.001	10.44 ± 4.45	13.46 ± 4.88	<0.001	7.33 ± 3.51	9.88 ± 4.20	<0.001
BMI (kg/m^2^), mean ± SD	24.25 ± 3.36	26.02 ± 3.63	<0.001	23.94 ± 3.28	25.78 ± 3.51	<0.001	24.44 ± 3.39	26.19 ± 3.70	<0.001
WC (cm), mean ± SD	82.25 ± 9.90	87.80 ± 10.28	<0.001	83.69 ± 10.03	89.40 ± 10.49	<0.001	81.32 ± 9.71	86.73 ± 10.00	<0.001
WHR, mean ± SD	0.88 ± 0.07	0.91 ± 0.07	<0.001	0.89 ± 0.07	0.93 ± 0.07	<0.001	0.87 ± 0.07	0.90 ± 0.07	<0.001
WHtR, mean ± SD	0.51 ± 0.06	0.55 ± 0.06	<0.001	0.50 ± 0.06	0.54 ± 0.06	<0.001	0.52 ± 0.06	0.56 ± 0.06	<0.001

*SD, standard deviation; RMB, renminbi; T2DM: type 2 diabetes; FBG, fasting blood-glucose; Hs-CRP, hypersensitive c-reactive protein; TG, triglyceride; TC, cholesterol; HDL-C, high-density lipoprotein cholesterol; LDL-C, low-density lipoprotein cholesterol; BFP, body fat percentage; VFI, visceral fat index; BMI, body mass index; WC, waist circumference; WHR, waist-to-hip ratio; WHtR, waist-to-height ratio*.

### Relationship Between Continuous BFP and Hypertension

**Figure 2:**
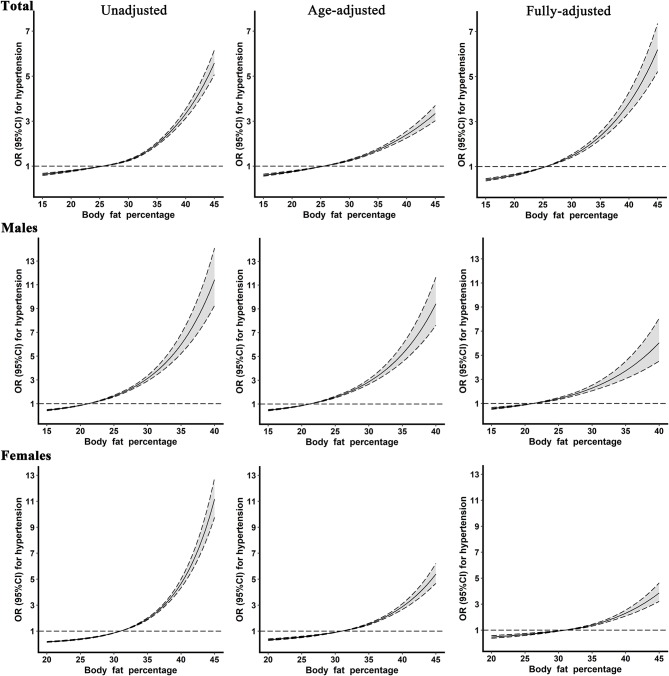
The restricted cubic spline for the relationship between body fat percentage levels and hypertension. Reference values for body fat percentage were set at the cutoff values of the first sex-specific quartiles. The fully adjusted model included age, gender (only in the total population), education level, average monthly individual income, marital status, smoking, alcohol consumption, physical activity, high-fat diet, more vegetable and fruit intake, family history of hypertension, type 2 diabetes, fasting blood glucose, hypersensitive c-reactive protein, triglyceride, cholesterol, high-density lipoprotein cholesterol, and low-density lipoprotein cholesterol.

Figure 2 presents restricted cubic spline curves with three knots located at the 25 (reference), 50, and 75 percentiles of BFP levels, respectively, to examine the relationships that linked continuous BFP with the hypertension in the total population, males, and females. As demonstrated, the risk of hypertension elevated steadily with continuously increasing BFP after adjusting for confounders. Also, dose–response relationships that linked BFP with the risk of hypertension were observed in both genders.

### Relationship Between Categorical BFP and Hypertension

As shown in Table 2, compared with participants in the lowest BFP quartile, the OR (95% CI) of the highest BFP quartile for hypertension was 3.31 (3.11, 3.53) in the total population, with a corresponding OR (95% CI) of 5.37 (4.83, 5.98), and 6.96 (6.37, 7.62) in males and females in model 1. After further adjustment of confounders, the OR (95% CI) of the highest BFP quartile for hypertension was 4.19 (3.69, 4.75), 3.30 (2.85, 3.83), and 2.66 (2.36, 2.99) in the total population, males, and females in model 3, respectively. Similarly, 1-SD increases in BFP were significantly associated with 87, 62, and 54% higher risks of hypertension in the total population, males, and females, respectively.

**Table 2:** Odds ratios (95% CIs) for hypertension according to BFP levels.

	**BFP quartiles**					**OR for 1-SD increment (95% CI)**
	**Q_**1**_**	**Q_**2**_**	**Q_**3**_**	**Q_**4**_**	***P*_**for**__**trend**_**	
**Total**						
Model 1	1.00 (ref.)	1.59 (1.49, 1.70)	1.67 (1.56, 1.78)	3.31 (3.11, 3.53)	<0.001	1.62 (1.58, 1.65)
Model 2	1.00 (ref.)	1.63 (1.53, 1.75)	1.66 (1.55, 1.77)	2.66 (2.49, 2.84)	<0.001	1.47 (1.44, 1.51)
Model 3	1.00 (ref.)	1.87 (1.70, 2.04)	2.64 (2.35, 2.97)	4.19 (3.69, 4.75)	<0.001	1.87 (1.78, 1.97)
**Males**						
Model 1	1.00 (ref.)	1.88 (1.68, 2.10)	3.03 (2.72, 3.38)	5.37 (4.83, 5.98)	<0.001	1.95 (1.87, 2.02)
Model 2	1.00 (ref.)	1.80 (1.60, 2.01)	2.83 (2.53, 3.15)	4.74 (4.25, 5.28)	<0.001	1.85 (1.78, 1.93)
Model 3	1.00 (ref.)	1.49 (1.29, 1.71)	2.21 (1.91, 2.54)	3.30 (2.85, 3.83)	<0.001	1.62 (1.53, 1.71)
**Females**						
Model 1	1.00 (ref.)	2.24 (2.04, 2.46)	3.42(3.12, 3.75)	6.96 (6.37, 7.62)	<0.001	2.19 (2.12, 2.26)
Model 2	1.00 (ref.)	1.77 (1.61, 1.95)	2.30 (2.09, 2.53)	3.74 (3.40, 4.12)	<0.001	1.71 (1.64, 1.77)
Model 3	1.00 (ref.)	1.50 (1.34, 1.69)	1.82 (1.62, 2.05)	2.66 (2.36, 2.99)	<0.001	1.54 (1.47, 1.61)

*Model 1: unadjusted. Model 2: adjusted for age. Model 3: further adjusted for gender (only in the total population), education level, average monthly individual income, marital status, smoking, alcohol consumption, physical activity, high-fat diet, more vegetable and fruit intake, family history of hypertension, T2DM, TG, TC, HDL-C, LDL-C, Hs-CRP, and FBG. BFP was divided into sex-specific quartiles (for total population: Q1: <25.5, Q2: 25.5–30.7, Q3: 30.7–35.1, Q4: ≥35.1; for males: Q1: <21.2, Q2: 21.2–24.6, Q3: 24.7–27.8, Q4: ≥27.9; for females: Q1: <31.0, Q2: 31.0–34.0, Q3: 34.1–36.8, Q4: ≥36.9)*.

### Comparison of BFP With Other Adiposity Measures

The ORs and 95% CIs for adiposity measures (BMI, WC, WHR, WHtR, and VFI) associated with hypertension were depicted in Figure S1. After adjusted potential confounders, similar to BFP, the risk of hypertension rose along with increasing adiposity measures quartile. The AUCs (95% CI) of BMI, WC, WHR, WHtR, VFI, and BFP were 0.642 (0.636, 0.647), 0.652 (0.646, 0.658), 0.641(0.635, 0.647), 0.667 (0.662, 0.673), 0.670 (0.664, 0.675), and 0.626 (0.620, 0.632) in the total population, respectively. BFP showed the lowest identifying power compared to other adiposity measures in the total population. However, after stratified by gender, BFP had the highest and second discriminatory capability for the risk of hypertension in females (AUCs: 0.696; 95% CI: from 0.689 to 0.703) and males (AUCs: 0.673; 95% CI: from 0.665 to 0.682), respectively (see Figure S2 and Table S1). In addition, the results suggested that VFI was the best discrimination index of hypertension risk in males, whereas the AUCs of VFI with BFP were not statistically significantly different.

## Discussion

This study was carried out in a large rural population-based sample, and the relationship between BFP and hypertension was evaluated. We found that the prevalent hypertension increased with rising BFP quartiles in rural areas of Henan province, and a considerably stronger association of BFP and hypertension prevalence in the total population and both genders was observed by binary logistic regression models. In addition, significant dose–response relationships between continuous BFP levels and risk of hypertension in the total population and both males and females were also revealed by restricted cubic spline models. Moreover, ROC curve analysis demonstrated that BFP had the highest discrimination power for hypertension risk in females.

The crude prevalence of hypertension in this study was 32.58% (32.94% for males and 32.35% for females, respectively). A study (24) performed in the general population of China reported that the prevalence of hypertension was 32.5% (33.7% for males and 31.9% for females, respectively). Additionally, a systematic analysis (25) showed that the prevalence of hypertension in Henan province was 33.5% (32.5% for males and 34.0% for females, respectively), and the rural areas seemed to have higher prevalent hypertension compared to urban areas (30.8 vs. 26.9%). These studies aforementioned showed that the rural areas of Henan province had a high prevalence. Therefore, this study was implemented in such a rural area and might be more meaningful for controlling the prevalence of hypertension.

In the present study, we found a significantly positive association between BFP and prevalent hypertension in the total population, males, and females, and the risk of hypertension progressively elevated with increasing BFP. Similar findings were also observed in other studies. Previous studies (19, 26) in Southern China reported that high BFP was concerned with hypertension prevalence, as well as systolic pressure and diastolic pressure. Besides, a prior study (27) in Anhui province indicated that hypertension prevalence in children aged 7–17 years also increased along with the increase of BFP. Furthermore, a recent cohort study demonstrated that BFP was an important predictor of incident hypertension (28). Our study indicated that compared to participants in the lowest BFP quartile, the ORs (95% CIs) of the highest BFP quartile were 3.30 (2.85, 3.83) in males and 2.66 (2.36, 2.99) in females after adjusting for confounders, which was comparable to these studies. In addition, the relationships between continuous BFP and prevalent hypertension in different genders were further analyzed in this study, and dose–response relationships of BFP with prevalent hypertension were also observed irrespective of genders. However, in contrast to the above studies, Hu L et al. reported a cross-sectional study with individuals aged 15 or older in whom BFP had significant correlation with prehypertension rather than hypertension in Jiangxi Province of China (29). A cohort study, which included 903 participants and conducted in African-Americans aged 18–65 years, also reported that BFP, as a total adiposity index, was not related to the risk of hypertension, with a corresponding OR (95% CI) of 1.01 (0.83, 1.23) (15). These inconsistencies might be due to different demographic characteristics such as racial, age ranges, survey locations, sample size or some potentially undetected factors of study population (14, 30). For instance, a study conducted in Houston demonstrated that the association of obesity with hypertension was altered with different race [30]. In that study, compared to other adiposity measures, BFP showed better discriminatory capability for the risk of hypertension in both females and males. Likewise, a study (14) conducted in Henan province suggested that BFP had larger AUCs than other adiposity measures including BMI, WC, WHtR, and VFI, which were similar to our results. However, BFP had the lowest identifying power in the total population, which might be explained by great gender differences. Therefore, the relationship between BFP and hypertension is worth using prospective and multicenter designs for further validation.

There are some biologically plausible metabolic mechanisms that could illustrate how the speculated BFP–hypertension pathway might work. Excess energy can be stored in adipose tissue when people ingest too much, and individuals with excess body fat deposition can secrete more inflammatory cytokines than subjects with normal weight (31). On the other hand, the degree of sympathetic activation is higher in those who have greater body fat (32), which may affect metabolic alterations (33). In addition, body fat can induce the occurrence of kidney disease and finally contribute to glomerulosclerosis (34). Furthermore, insulin resistance is able to stimulate and increase the reabsorption of sodium in those with more body fat distribution (35) and the increase of renal tubular sodium reabsorption has a certain inhibitory effect on the pressure natriuretic and plays an important role in the development of hypertension. To date, the potential mechanisms have not been thoroughly cleared yet; therefore, further studies are needed to illuminate the exact mechanisms.

Despite this study including a relatively large representative sample size of the Chinese rural population, as well as adjusting a mass of confounders, some limitations also existed. First, in this study, BFP was derived from bioelectrical impedance methods. Although a bioelectrical impedance method is not as accurate as the gold standard, it is more suitable for a large-scale epidemiological investigation. Second, selection bias as well as residual confounding such as hemoglobin A1c (HbA1c) and other related biomarkers cannot be thoroughly ruled out, although we adjusted for a large number of covariates to minimize the effects of confounders. In addition, the cross-sectional study elucidated that a conclusive cause-and-effect relationship is limited. Therefore, further researches are warranted to confirm the extensibility of our results.

## Conclusions

BFP was significantly positively associated with the prevalence of hypertension in the Chinese rural population. These findings indicated that BFP ≥ 24.85 in males and BFP ≥ 34.95 in females will increase the risk of hypertension. BFP may play a key role in early identification of hypertension, especially in females, and could provide some useful information to clinicians to remind patients of hypertension prevention via controlling BFP within a reasonable range, which, in the long run, may improve the health level and further reduce the disease burden in Chinese rural residents.

## Data Availability Statement

The raw data supporting the conclusions of this article will be made available by the authors, without undue reservation, to any qualified researcher.

## Ethics Statement

The studies involving human participants were reviewed and approved by Zhengzhou University Life Science Ethics Committee. The patients/participants provided their written informed consent to participate in this study.

## Author Contributions

RL and ZT analyzed the data and wrote the manuscript. CW and RB designed the study. RL, ZT, YanhW, XL, RT, YanW, XD, YiW, DW, HT, ZM, LL, and WH conducted the collection of the data. XL corrected the manuscript. All authors read and approved this version of the article.

### Conflict of Interest

The authors declare that the research was conducted in the absence of any commercial or financial relationships that could be construed as a potential conflict of interest.
