# Supplementary material for: The Association of Body Fat Percentage With Hypertension in a Chinese Rural Population: The Henan Rural Cohort Study
Source: Front Public Health. 2020 Mar 20;8:70. doi: 10.3389/fpubh.2020.00070 (PMC7103629; doi:10.3389/fpubh.2020.00070)
Supplement: Supplementary file 1 [file Data_Sheet_1.PDF]

1. Supplementary Figures and Tables

Figure legends:

Figure S1. Odds ratios (95% CIs) for multiple adiposity indices associated with hypertension

CI: confidence interval; BMI: body mass index; WC: waist circumference; WHR: waist to hip ratio; WHtR: waist to height ratio; VFI: visceral fat index. These models were adjusted for age, gender (only in the total population), education level, average monthly individual income, marital status, smoking, alcohol consumption, physical activity, high fat diet, more vegetables and fruits intake and, family history of hypertension, type 2 diabetes, fasting blood-glucose, hypersensitive c-reactive protein, triglyceride, cholesterol, high density lipoprotein cholesterol, and low density lipoprotein cholesterol.

Figure S2. Receiver operating characteristic curves for multiple adiposity indices in identifying hypertension

BMI: body mass index; WC: waist circumference; WHR: waist to hip ratio; WHtR: waist to height ratio; VFI: visceral fat index; BFP: body fat percentage.

1. 1 Supplementary Figures

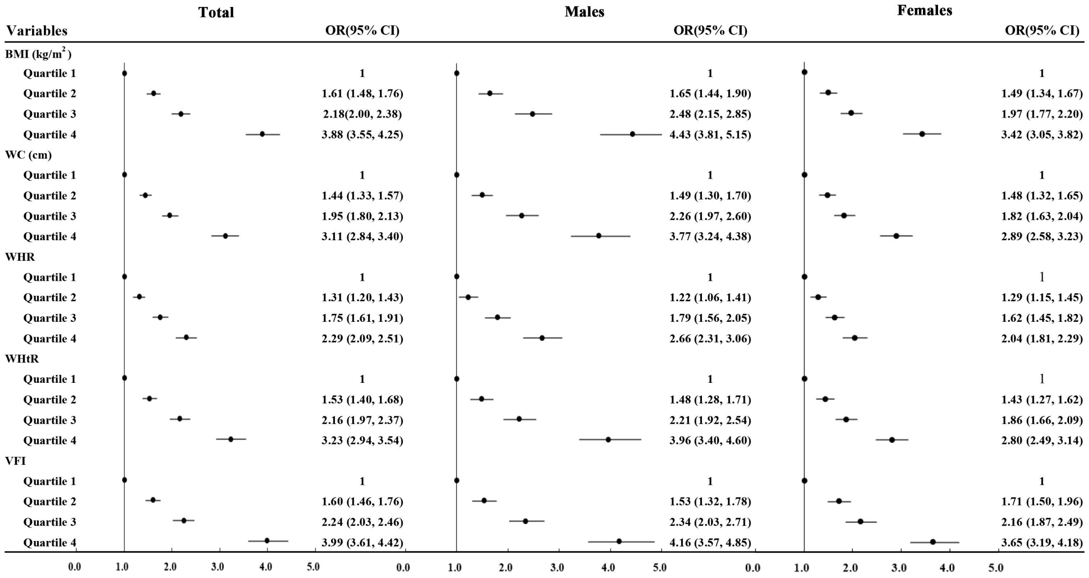

Figure S1. Odds ratios (95% CIs) for multiple adiposity indices associated with hypertension. CI: confidence interval; BMI: body mass index; WC: waist circumference; WHR: waist to hip ratio; WHtR: waist to height ratio; VFI: visceral fat index. These models were adjusted for age, gender (only in the total population), education level, average monthly individual income, marital status, smoking, alcohol consumption, physical activity, high fat diet, more vegetables and fruits intake and, family history of hypertension, type 2 diabetes, fasting blood-glucose, hypersensitive c-reactive protein, triglyceride, cholesterol, high density lipoprotein cholesterol, and low density lipoprotein cholesterol.

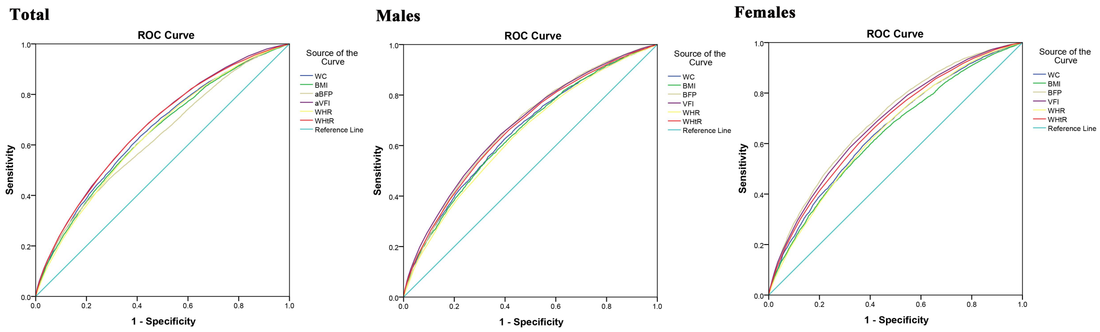

Figure S2. Receiver operating characteristic curves for multiple adiposity indices in identifying hypertension. BMI: body mass index; WC: waist circumference; WHR: waist to hip ratio; WHtR: waist to height ratio; VFI: visceral fat index; BFP: body fat percentage.

1. 2 Supplementary Table

Table S1 AUC (95% CI) of adiposity indices identifying for hypertension

	AUC	*95% CI*	Cut-off value	Sensitivity	Specificity	Youden index
**Total**						
BMI	0.642	(0.636, 0.647)	24.96	0.60	0.60	0.20
WC	0.652	(0.646, 0.658)	84.25	0.63	0.59	0.22
WHR	0.641	(0.635, 0.647)	0.89	0.65	0.55	0.20
WHtR	0.667	(0.662, 0.673)	0.54	0.61	0.63	0.24
VFI	0.670	(0.664, 0.675)	9.50	0.60	0.64	0.24
BFP	0.626	(0.620, 0.632)	33.45	0.47	0.71	0.18
**Males**						
BMI	0.650	(0.641, 0.659)	25.33	0.54	0.67	0.21
WC	0.654	(0.645, 0.663)	86.25	0.62	0.60	0.23
WHR	0.639	(0.630, 0.649)	0.91	0.63	0.57	0.20
WHtR	0.667	(0.658, 0.676)	0.52	0.66	0.59	0.25
VFI	0.677	(0.668, 0.686)	11.50	0.65	0.62	0.26
BFP	0.673	(0.665, 0.682)	24.85	0.66	0.60	0.26
**Females**						
BMI	0.637	(0.629, 0.644)	24.97	0.61	0.58	0.20
WC	0.652	(0.645, 0.660)	83.45	0.63	0.59	0.22
WHR	0.646	(0.639, 0.653)	0.88	0.65	0.56	0.21
WHtR	0.672	(0.665, 0.679)	0.54	0.67	0.58	0.25
VFI	0.684	(0.677, 0.692)	8.50	0.58	0.68	0.26
BFP	0.696	(0.689, 0.703)	34.95	0.61	0.67	0.28

Abbreviations: AUC: area under the curve; CI: confidence interval; BMI: body fat index; WC: waist circumference; WHR: waist to hip ratio; WHtR: waist to height ratio; VFI: visceral fat index; BFP: body fat percentage.
